# Bimetallic AgPd/UiO-66 Hybrid Catalysts for Propylene Glycol Oxidation into Lactic Acid

**DOI:** 10.3390/ma13235471

**Published:** 2020-11-30

**Authors:** Sergey Ten, Viktoriia V. Torbina, Vladimir I. Zaikovskii, Sergei A. Kulinich, Olga V. Vodyankina

**Affiliations:** 1 Laboratory of Catalytic Research, Tomsk State University, 36 Lenin Ave., 634050 Tomsk, Russia; rip_richard@mail.ru (S.T.); ms.itory@mail.ru (V.V.T.); 2 Boreskov Institute of Catalysis, 5, Lavrentiev Ave., 630090 Novosibirsk, Russia; viz@catalysis.ru; 3 Research Institute of Science & Technology, Tokai University, Kanagawa 259-1292, Japan; skulinich@tokai-u.jp; 4 School of Natural Sciences, Far Eastern Federal University, 690091 Vladivostok, Russia

**Keywords:** metal-organic framework, bimetallic nanoparticles, silver, palladium, cascade propylene glycol oxidation, lactic acid

## Abstract

Different methods (the wetness impregnation of Ag and Pd precursors dissolved in water or acetonitrile solution, and the double solvent impregnation technique) were employed to immobilize Ag–Pd nanoparticles (NPs) into the pores of the microporous zirconium-based metal-organic framework known as UiO-66. The obtained materials were characterized by using nitrogen adsorption-desorption at −196 °C, powder X-ray diffraction, UV-Vis diffusion reflectance spectroscopy, and transition electron microscopy measurements. Special attention was paid to the acid and redox properties of the obtained materials, which were studied by using temperature-programmed desorption of ammonia (TPD-NH_3_) and temperature-programmed reduction (TPR-H_2_) methods. The use of a drying procedure prior to reduction was found to result in metallic NPs which, most likely, formed on the external surface and were larger than corresponding voids of the metal-organic framework. The formation of Ag–Pd alloy or monometallic Ag and Pd depended on the nature of both metal precursors and the impregnation solvent used. Catalytic activity of the AgPd/UiO-66 materials in propylene glycol oxidation was found to be a result of synergistic interaction between the components in AgPd alloyed NPs immobilized in the pore space and on the external surface of UiO-66. The key factor for consistent transformation of propylene glycol into lactic acid was the proximity between redox and acid-base species.

## 1. Introduction

Intensification of catalytic processes for selective conversion of biomass derivatives into value-added chemicals by catalyzing multistep transformations over single multifunctional catalyst has been attracting increasing attention during the past two decades due to “green” chemistry and cost effectiveness requirements [1]. Propylene glycol (PG) conversion into lactic acid (LA) under mild conditions is one of the important processes related to production of monomer for biodegradable plastics from bio-renewable feedstock [2]. This process consists of three consecutive steps, including the oxidation of the secondary hydroxyl group of PG with the formation of hydroxyacetone (HA) for the first step and HA oxidation in to methylglyoxal (MGO) during the second step. During the last step, MGO is converted into lactic acid (LA) via an intramolecular Cannizarro reaction [3]. Redox active sites catalyze the two initial steps. The noble metal nanoparticles on different supports such as carbon, alumina, silica, etc. [2] are often used as catalysts for the oxidation of alcohols. Alkaline media catalyzes the last step of the process. Intramolecular Cannizarro reaction can also be catalyzed by using solid Lewis acid [4]. A combination of redox species and solid Lewis acid sites allows for converting PG to LA in neutral media in one reactor without additional separation or purification of semi-products. Thus, the application of a multifunctional catalyst in PG oxidative conversion makes it both “green” and cost effective.

The best candidates for the role of such a multifunctional catalyst comprise solid materials with active sites of different natures. Metal-organic frameworks (MOFs) are promising materials for creating hybrid composites due to both the unique properties of the support and high concentration of accessible active sites that are able to catalyze redox and acid-base reactions. In literature, there are different approaches towards the synthesis of composite materials using MOF, including the preparation of semi-conductive materials that are promising for photocatalytic and electrocatalytic applications [5,6]. High pore volume and cavities with a strictly defined size of MOFs allow for stabilizing nanoparticles (NPs) with diameters below 5 nm that will be available for reagents due to the framed structure of the MOFs [7,8].

Noble metal NPs (Au, Pt, Pd, etc.) supported on traditional inorganic materials, e.g., silica, alumina, and active carbon are well known as active catalysts for liquid-phase oxidation of various kinds of organic compounds [9,10]. At the same time, the application of silver and silver-doped NPs as catalysts/electrocatalysts for mild oxidation [11,12,13,14,15,16], gas-phase hydrogenation/dehydrogenation [17,18], methanolysis [19,20], reduction [21], and hydrogen generation [22,23,24] has attracted increasing attention. In addition to obvious economic benefits, the advantage of Ag-containing catalysts is the partial oxidation reaction that is not accompanied by C–C bond cleavage reactions, as was shown in work [25] for selective glycerol oxidation into dihydroxyacetone on Ag-Pd/C catalysts. However, the control of size and shape of Ag NPs is quite a difficult task in comparison with Au, Pd, and Pt NPs [26].

Porous zirconium terephtalate UiO-66 is a promising active support for metal NPs owing to its large surface area, high pore volume, as well as its high thermal, chemical, and aqueous stability [27,28]. The large fraction of accessible Zr^4+^ active sites for UiO-66 make it a highly active catalyst for Lewis acid-catalyzed reactions [29,30,31].

There are a number of publications regarding the synthesis of Ag/MOF, Pd/MOF, and AgPd/MOF hybrid materials; however, MOF is only used as support in most cases and has a minor effect on the formation and structural features of metal/bimetal NPs localized on its external surface. Ag/MOFs have found applications in gas- and liquid-phase reactions such as conversion of terminal alkynes into propiolic acid with CO_2_ [32], CO oxidation [33], dehydrogenation of formic acid [34], styrene oxidation [35], catalytic hydrolysis of ammonia borane [36], and as electrochemical sensors [37]. Some methods used to prepare Ag/MOF materials are described in the literature [24,25,26,27,29,38,39,40,41,42,43,44,45,46]. In Refs. [30,31], a simple adsorption of freshly formed Ag and Au–Ag NPs on porous cobalt salicylate (Co-MOF) and ZIF-8, respectively, from the colloidal solution were employed. However, the size of NPs exceeded the pore size of the MOFs used, indicating their localization on the external surface of the support and/or partial decomposition of the MOF structure. The one-pot simultaneous synthesis of Ag NPs, and MOF (UiO-66) employed by Li et al. [27] has some restrictions connected with a selection of the optimal reaction conditions. Despite its obvious simplicity, impregnation of MOF with an excess level of an aqueous solution of silver nitrate and the subsequent reduction of silver cations to Ag^0^ NPs did not allow for controlling the size distribution and localization of Ag NPs directly inside the porous space of the MOF [26,29,34,35,36]. An impregnation of MOF with acetonitrile solution of AgNO_3_ and subsequent Ag reduction by using different methods led to preferential formation of Ag NPs inside the pores of the MOFs [24].

Recently, Aijaz et al. [47] developed a so-called “double solvent” (DS) approach for the immobilization of ultrafine Pt NPs directly inside the pores of MIL-101 without triggering aggregation of NPs on the external surface. Under this method, a large amount of hydrophobic solvent was used to disperse the MOF powder. Then the required amount of aqueous solution of metal precursor with a volume set equal to or below the pore volume of MIL-101 was added to the suspension. Due to capillary forces that originated from the hydrophilic nature of Cr-MIL-101 pores, the limited amount of aqueous solution was completely absorbed into the MOF cavities. In contrast, in the conventional single solvent impregnation process, a large amount of solution of precursors was deposited on the outer surface of the MOF. After impregnation, n-hexane was decantated, and the materials were dried at different temperatures followed by the reduction of metals through the standard procedures with H_2_ or NaBH_4_. The DS approach proved to be an effective method to immobilize different single-metal and bimetallic NPs directly inside the pores of mesoporous MIL-101 MOFs with a very large surface area and pore volume using different functional groups [48,49,50,51,52]. However, when Zhu et al. [53] tried to encapsulate Au NPs into the microporous UiO-66 by using the DS method, the formation of NPs with a diameter exceeding the size of UiO-66 cages was confirmed by the XRD data. This challenge could be the result of a much higher relation between the outer and inner surfaces of microporous MOFs, which have a smaller pore size and volume than those in the mesoporous MIL-101. After impregnation, a drying step may lead to redistribution of components and diffusion of metal sources out of the MOF pores with water evaporation, followed by the formation of NPs on the external surface.

One of the main challenges related to the use of silver comes from its high reduction ability, especially under UV-vis irradiation [54]. The latter is known to lead to the formation of monometallic Ag NPs rather than those of bimetallic alloys.

Here, we report the synthesis of bimetallic AgPd hybrid materials by means of the DS approach with a one-pot reduction without drying. Moreover, a solution of metal precursors in acetonitrile (MeCN) instead of water is used during the incipient wetness impregnation. This approach prevents the redistribution of metal precursors and formation of silver NPs on the external surface of the MOF during drying. After successful addition of redox sites in the form of AgPd NPs, the obtained materials are used as a bifunctional heterogeneous catalyst in a propylene glycol oxidation process.

## 2. Experimental Section

### 2.1. Preparation of UiO-66

UiO-66 was synthesized by using the solvothermal method according to a previously reported procedure [55]. Equimolar amounts (3 mmol) of terephthalic acid and ZrO(NO_3_)_2_·2H_2_O were dispersed in DMF (60 mL), then hydrochloric acid (99 mmol) was added. The mixture was placed into a Teflon-lined stainless steel autoclave and heated at 120 °C for 24 h. The obtained white solid was washed with DMF and ethanol. The structure of the UiO-66 material was confirmed by using XRD and Fourier transform IR-spectroscopy (FT-IR) data (Figures S1 and S2 in Supporting Information (SI), respectively), with the BET surface area and micropore volume found to be 1298 m^2^/g and 0.50 cm^3^/g∙nm, respectively. Prior to the characterization and encapsulation of the metal NPs, the obtained UiO-66 was activated at 150 °C under vacuum.

### 2.2. Synthesis of AgPd@UiO-66

#### 2.2.1. Incipient Wetness Impregnation Method (IWI)

Typically, the calculated amounts of silver and palladium nitrates were dissolved in 1.5 mL of water. The prepared impregnation solution was added to 1 g of UiO-66 dropwise under stirring. The impregnated material was dried at room temperature overnight. The reduction of Ag and Pd was carried out in a flow of H_2_ in argon (10% H_2_(vol)) at 200 °C over a 2 h period.

The impregnation with AgPd NPs from acetonitrile solution was performed by using the same method except for the preparation of the impregnation solution. Acetonitrile impregnation solution was prepared by mixing of the required amounts of silver nitrate and palladium acetylacetonate in 1.9 mL of MeCN. The obtained solution was added to 1 g of UiO-66 dropwise under stirring. Further steps were similar to those mentioned above.

#### 2.2.2. Double Solvent Approach (DS)

A total of 1 g of activated UiO-66 was suspended in 40 mL of anhydrous n-heptane acting as a hydrophobic solvent. The obtained mixture was sonicated for 30 min. After that, 0.50 mL of hydrophilic aqueous solution of silver and palladium nitrates was added dropwise under vigorous stirring. After the stirring of the resultant suspension for 1 h, the 62%wt. aqueous solution of hydrazine was added dropwise. The mixture was stirred for 1 h. The solid was isolated from n-heptane by using decantation. The prepared material was dried at 150 °C with a heating rate of 3°/min that was sufficient for the removal of solvent and other residual components after reduction (Figure S3 in the SI).

### 2.3. Characterization

Textural characteristics of UiO-66 and the hybrid catalysts were determined from nitrogen adsorption isotherms (−196 °C; 3Flex instrument, Micromeritics, Norcross, GA, USA). Prior to characterization, the samples were degassed under vacuum at 90 °C during 1 h and at 150 °C during 5 h. The Horvath-Kawazoe sphere pore geometry method was used to calculate the pore size distribution. Powder X-ray diffraction (XRD) patterns were collected using the XRD-6000 diffractometer (“Shimadzu”, Kyoto, Japan) with a monochromatized Cu Kα radiation (λ = 1.5418 Å) in the range of 2θ angles of 5–50° with a scanning rate of 2°/min. The identification of the phase composition was carried out with the PCPDFWIN database and the PowderCell 2.4 program (Kraus W. and Noize G., Berlin, Germany). Further profile analysis was done using Jana2006 software [56]. FT-IR spectra were taken using a SCIMITAR FTS 2000 spectrometer (DIGILAB, Hopkinton, MA, USA). Hybrid materials were also characterized by employing transmission electron microscopy (TEM) and energy dispersive X-ray spectroscopy (EDS) using the JEM-2200FS microscope (JEOL, Akishima, Japan) with a resolution of 0.1 nm at an accelerating voltage of 200 kV, and UV-Vis DRS using an Evolution 600 Thermo Scientific spectrometer (ThermoFisher Scientific, Waltham, MA, USA) with MgO. Morphology of the samples prepared was studied using the scanning electron microscope VEGA 3 SBH, Tescan (Brno, Czech) with SE and BSE detectors at an accelerating voltage of 5 kV.

Acid properties of UiO-66 and hybrid materials were determined by measuring the temperature-programmed desorption of NH_3_ (NH_3_-TPD) using chemisorption system Micrometrics AutoChem HP 2950 (Norcross, GA, USA). Prior to the measurements, the samples were activated at 300 °C in He atmosphere (30 mL/min). The sample was saturated with dried NH_3_ at 50 °C. The desorption proceeded at a heating rate of 10 °C/min from 50 °C to 350 °C. Temperature-programmed reduction measurements (TPR) were carried out using the chemisorption system Micrometrics ChemiSorb 2750 (Norcross, GA, USA) with a 10 mL min^−1^ flow of H_2_/Ar (10% H_2_) and a heating rate 2 °C/min. Prior to the experiment, 100 mg of the sample were pretreated at 250 °C in air flow for 20 min and then cooled down to room temperature. DTA/TGA data were collected with NETZSCH STA 449F1 Jupiter coupled mass-spectrometer QMS 403D Aeolos (heating 10 °C min^-1^ from 25 °C to 600 °C, 80% air/Ar, Al_2_O_3_ crucible, (NETZSCH-Gerätebau GmbH, Selb, Germany).

### 2.4. Catalytic Tests

Typically, 20 mL of 0.3 M propylene glycol (PG) solution was added into the round bottom glass reactor, then 70 mg of catalyst was added. After that, the reactor was purged with oxygen twice and finally filled with oxygen (P(O_2_) = 3 bar). Then the reactor was heated up to the desired temperature using an oil bath equipped with thermocouple. After the catalytic experiment, the reaction mixture was separated by using centrifugation. PG conversion, methylglyoxal (MGO), formaldehyde, and hydroxyacetone (HA) selectivities were determined by using GC, while lactic acid (LA), acetic acid (AA), and formic acid (FA) selectivities were determined by HLPC.

## 3. Results and Discussion

### 3.1. Comparison of Synthetic Methods

In the present work, three methods of preparation of monometallic Ag@UiO-66, Pd@UiO-66, and bimetallic AgPd@UiO-66 (wetness impregnation by silver and palladium precursors dissolved in water (or acetonitrile) solution and double solvent impregnation technique) hybrids with variations of metal sources, the nature of solvents, and reducing agents were employed (Table 1).

**Figure 1:**
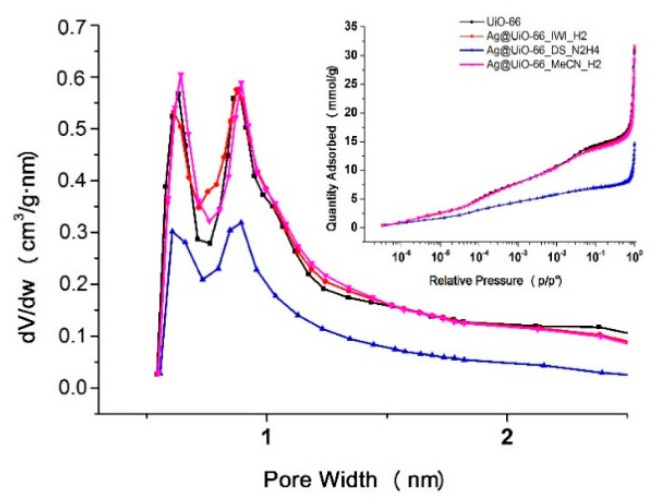
Pore size distributions and low-temperature N_2_ adsorption-desorption isotherms (in box) of UiO-66 (black) and Ag@UiO-66 samples prepared by IWI_H_2_ (red), MeCN_H_2_ (magenta), and DS_N_2_H_4_ (blue).

In order to compare the effect of the methods used, we prepared three monometallic Ag-containing samples with 1%wt. Ag content. Figure 1 represents the adsorption-desorption isotherms as well as the pore size distributions for the samples prepared. Table 2 summarizes the textural characteristics of the samples.

The initial UiO-66 sample was characterized by using bimodal micropore size distribution with two maxima at 0.6 and 0.9 nm according to the Horvath-Kawazoe method, and the BET surface area was 1298 m^2^/g. The texture characteristics were close to that of the UiO-66 material prepared by Cavka [27]. The Ag introduction into UiO-66 porous structure by employing wet impregnation method with the subsequent drying and H_2_ reduction at 200 °C during 2 h did not change the BET surface area for WI_H_2_ and MeCN_WI_H_2_ samples in practice (Table 2).

For the Ag@UiO-66_ DS_N_2_H_4_ sample prepared by using the double solvent technique with hydrazine reduction, the decrease of BET surface area by 24.2 %rel. and a total micropore volume by 22.4 %rel. was observed. The decrease of height of maxima on the pore distribution curve and slight shift to lower values for this sample (Figure 1, blue curves) was associated with the filling of pores of UiO-66 by Ag clusters. Thus, the immobilization of Ag clusters in the micropores of UiO-66 was observed for the sample prepared using the DS technique.

Diffusion reflectance UV-Vis spectroscopy data are a useful tool to study the highly dispersed silver species (their size, shape, presence, and structure of adsorption layers) [57,58]. According to the Mie’s theory, a location of plasmon resonance band depends on the shape and size of Ag NPs [59,60].

**Figure 2:**
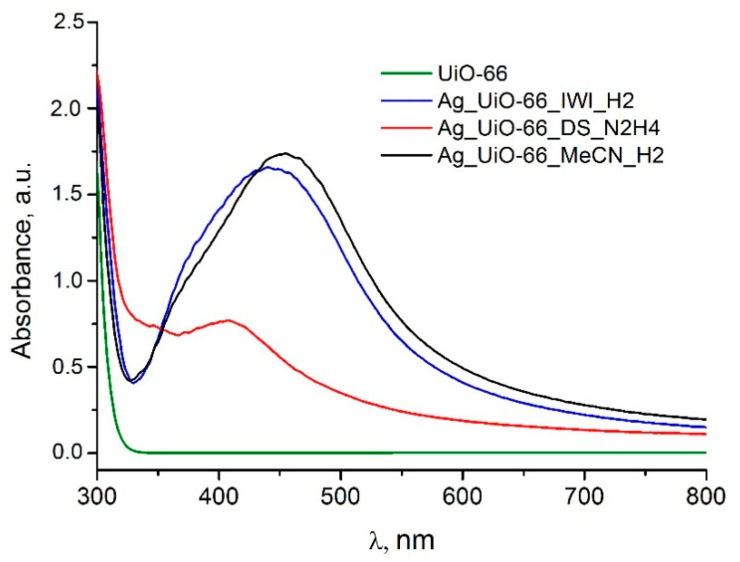
UV-Vis DR spectra of UiO-66 (green) and Ag@UiO-66 prepared by IWI_H_2_ (blue), MeCN_H_2_ (black), and DS_N_2_H_4_ (red).

Figure 2 shows the UV-Vis DR spectra for the samples prepared. The intensive band in the range of 200–300 nm of both pure UiO-66 and Ag-containing UiO-66 is attributed to absorption of UV radiation by the benzene ring of terephthalate ligand and ligand-to-zirconium charge transfer in the UiO-66 according to Ref. [61]. The UV-Vis DR spectra for the samples IWI_H_2_ and MeCN_IWI_H_2_ revealed a broad absorption band with a maximum at ~450 nm and a shoulder at 380 nm (Figure 2). The broad absorption band is caused by the overlapping of three absorption peaks. The first one with the maximum at 370–380 nm can be assigned to the resonance absorption of small Ag clusters. The band at 410 nm corresponds to Ag NPs with sizes of ~5–10 nm, and the one at 450 nm may be associated with the resonance absorption of aggregates of small Ag clusters or large Ag NPs [62,63]). This fact indicates that regardless of the solvent chosen, the incipient wetness impregnation and a high-temperature reduction with H_2_ lead to the formation of silver NPs with the sizes larger than the diameter of cages in the UiO-66 structure (0.6 and 0.9 nm, respectively [64]). In the spectrum for the DS_N_2_H_2_ sample, the intensity of the plasmon resonance band is much lower in comparison with those for the samples prepared by IWI, and its maximum is shifted towards the shorter wavelength region at 410 nm (Figure 2), indicating the formation of smaller Ag NPs. A shoulder at 300–320 nm can be attributed to the presence of Ag_3_ and Ag_4_ clusters, demonstrating the fingerprint absorptions at 303 and 320 nm, respectively [65]. A low intensity absorption at 350 nm can be assigned to the transitions in the small charged silver clusters (Ag_4_^2+^), as was shown for Ag-containing LTA zeolites [66].

**Figure 3:**
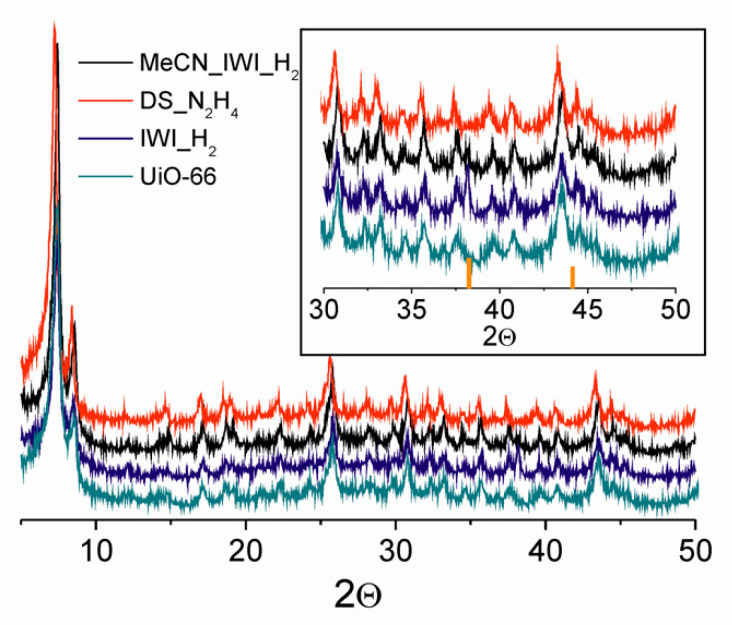
XRD pattern of UiO-66 and Ag@UiO-66 prepared by different methods. Orange bars correspond to reflexes of (111) and (200) crystal planes of zero-valent Ag FCC crystal.

The formation of crystalline Ag NPs with a diameter above 3 nm in the IWI_H_2_ and MeCN_IWI_H_2_ samples was also confirmed by the XRD data (Figure 3).

At the same time, only reflections of the UiO-66 structure were observed in the case of the sample prepared by using the double solvent approach. It is noteworthy that in all cases after immobilization of 1 %wt. of metal NPs, the structure of the MOF remained intact. Table S1 shows the data of close inspection of XRD patterns using the Jana2006 software. The amount of metallic Ag phase did not exceed 0.2%wt., which is consistent with the texture results and UV-Vis DR data on the immobilization of Ag clusters into the porous space of the MOF.

To investigate the effect of Pd addition to Ag, the bimetallic samples with 1%wt. total metal loading and Ag/Pd molar ratio of 1/1 were prepared by using different methods. Figure S4 shows the corresponding UV-Vis DR spectra. The presence of Pd in the bimetallic NPs is known to suppress the surface plasmon absorption band of Ag in the visible light region [67]. The sample prepared using the MeCN solution of metal sources with different natures exhibits the surface plasmon resonance band at 400 nm, thereby indicating the formation of some amount of monometallic Ag NPs located on the support surface. The formation of alloyed Ag–Pd NPs and/or small metal NPs inside the UiO-66 pores was observed based on the XRD and UV-vis DRS data for the cases of IWI_H_2_ and DS_N_2_H_4_ AgPd@UiO-66 samples. A dissipation in the visible range for all samples studied (Figure S4) can be connected with the effect of the metal–semiconductor/insulator grain boundary [68].

**Figure 4:**
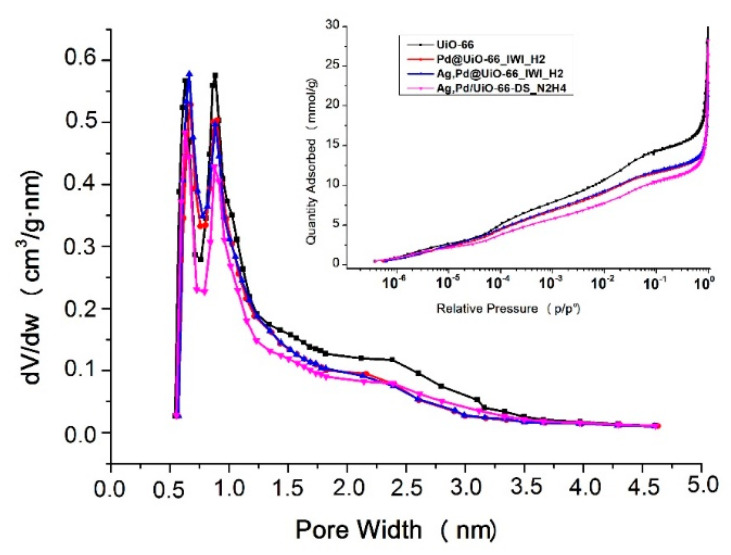
Pore size distribution and low-temperature N_2_ adsorption isotherms of UiO-66 (black), Pd@UiO-66_IWI_H_2_ (red) and AgPd@UiO-66 samples prepared by IWI_H_2_ (blue) and DS_N_2_H_4_ (magenta).

Figure 4 shows the data of low-temperature nitrogen adsorption-desorption along with the pore size distributions for bimetallic samples in comparison with the initial UiO-66 and monometallic Pd@UiO-66 samples.

Changes in the isotherm position were observed for all metal-containing samples that showed partial filling of porous space of UiO-66 by metal/bimetallic clusters. The introduction of Pd and AgPd NPs led to a decrease in the specific surface area and pore volume as compared with the pristine UiO-66 (Table 3), these variables remained most clear for the DS sample. Specific surface area and pore volume decreased by 24% in the latter case.

These data indicate the formation of highly dispersed metal NPs directly inside the pores of the host framework in the DS_N_2_H_4_ sample. A similar phenomenon was observed for the successfully immobilized Ag NPs in the cages of mesoporous MIL-101(Cr) [23]. Partial filling of the cages was more pronounced for the DS_N_2_H_4_ sample.

**Scheme 1:**
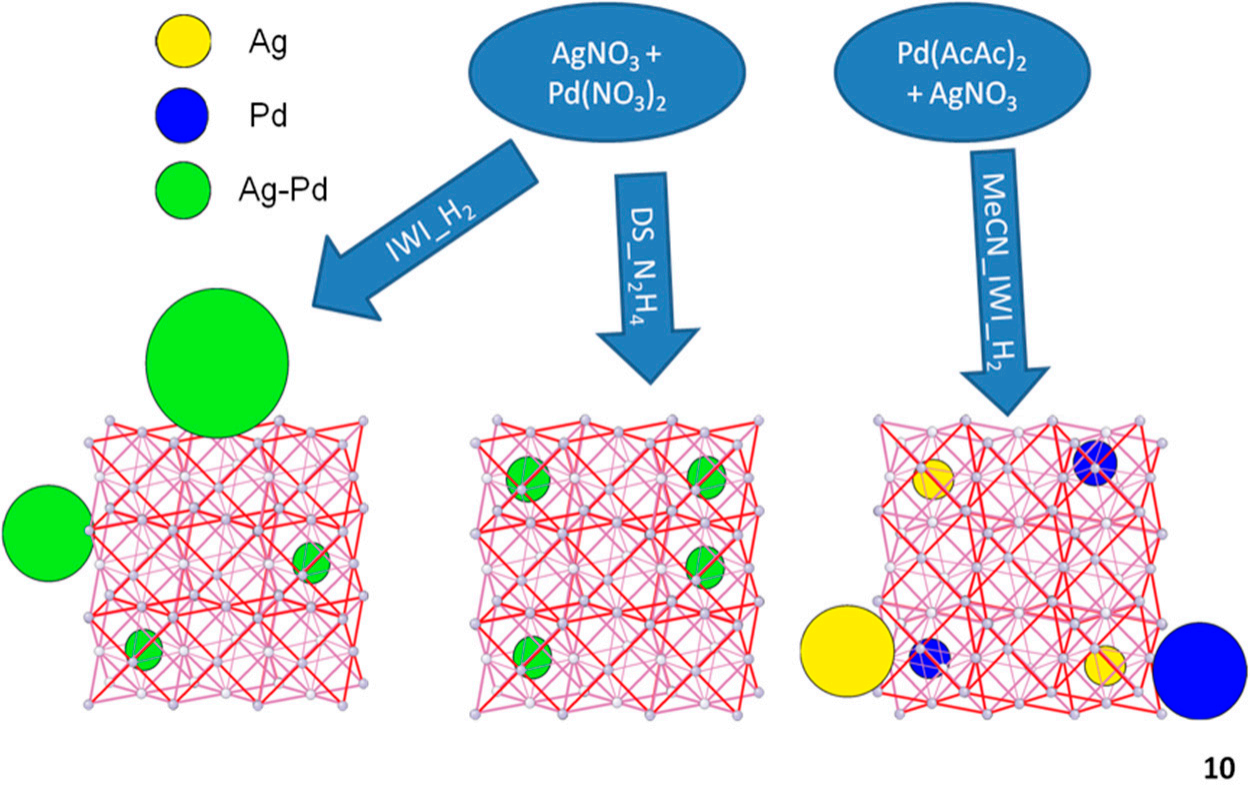
Structure of AgPd@UiO-66 materials prepared by using different methods.

Given the results of catalysts characterization, the schematic representation of the structure of AgPd@UiO-66 prepared by different methods can be proposed (Scheme 1). Only the DS_N_2_H_4_ method provides the successful immobilization of Ag and Ag–Pd bimetallic NPs in the UiO-66. However, for practical applications, higher metal loading can be required. Moreover, the increase in the metal content usually leads to the agglomeration of NPs and/or decomposition of the MOF structure. The DS_N_2_H_4_ method of NPs immobilization inside the UiO-66 was chosen for further investigation of the effect of metal loading.

### 3.2. Acid and Redox Properties

**Figure 5:**
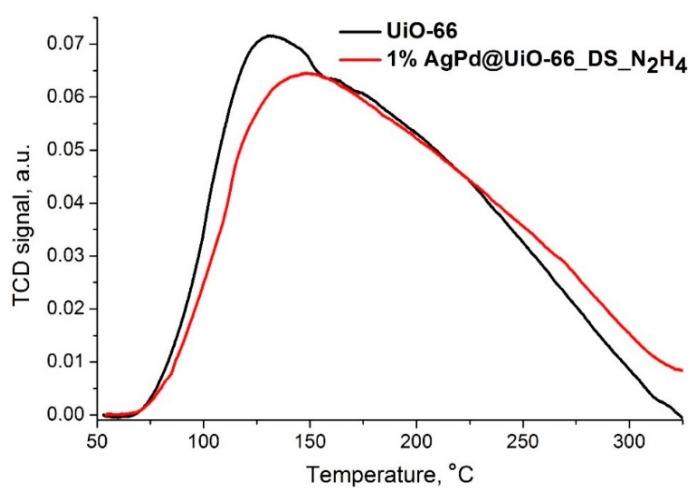
NH_3_-TPD profile of UiO-66 (black) and 1% AgPd@UiO-66 prepared by using DS_N_2_H_4_ (red).

The remaining accessibility of Zr active sites after metal immobilization is an important point for application of the synthesized hybrid material as a bifunctional catalyst. Figure 5 shows the NH_3_-TCD spectra of the pristine UiO-66 and 1% AgPd@UiO-66 sample prepared by using the DS_N_2_H_4_ method.

A slight decrease in the weak Lewis acidity [69] was observed for the metal-loaded sample in comparison with the pure UiO-66, although the total acidity was almost unchanged (Table 3). The calculated amount of zirconium Lewis acid sites (Table 3) was close to that obtained by Vermoortele et al. [21] for the UiO-66 prepared with trifluoroacetic acid as a modulator and HCl as a crystallizing agent, and corresponded to slightly less than two accessible active sites per the hexanuclear cluster Zr_6_O_4_(OH)_2_.

Redox properties of 1% AgPd@UiO-66 samples prepared by using different methods were investigated by using the H_2_-TPR technique. Due to the lower thermal stability of MOFs in comparison with inorganic supports, the conventional method of H_2_-TPR measurements at high temperatures could not be applied. Therefore, although Ag and Pd were reduced at relatively low temperatures [70,71], the oxidative treatment at 500 °C was not found to be appropriate. Thus, the low-temperature oxidative activity was studied. The samples were pretreated at 250 °C in an airflow. Moreover, the reduction was carried out at a lower heating rate in order to avoid the diffusion limitations for the microporous materials [72].

**Figure 6:**
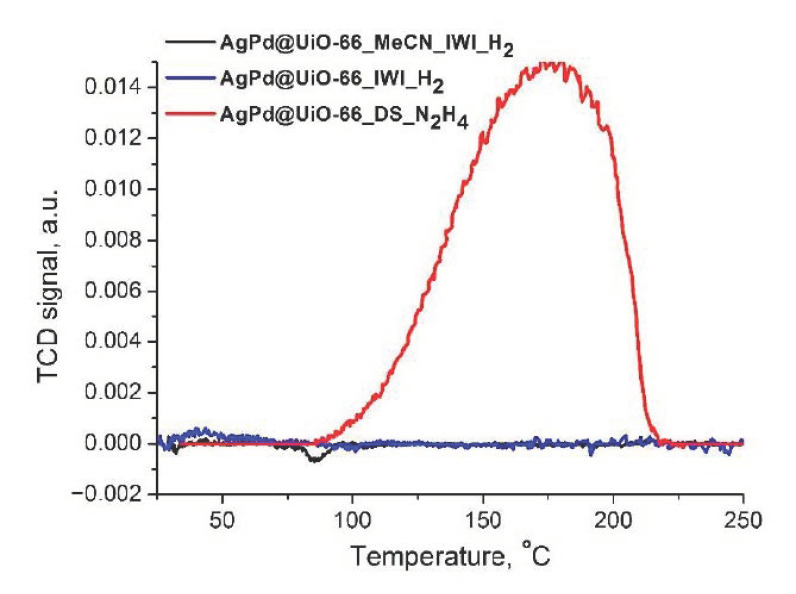
H_2_-TPR profiles of 1% AgPd@UiO-66 prepared by using DS_N_2_H_4_ (red), IWI_H_2_ (blue), and MeCN_IWI_H_2_ (black).

Figure 6 shows the corresponding H_2_-TPR profiles.

A negative peak in the profile of the MeCN_IWI_H_2_ sample is a common feature for Pd-containing materials and results from the release of hydrogen that was adsorbed on the surface of Pd NPs at sub-ambient temperatures in the form of Pd β-hydride [73]. According to Khan et al. [74], the addition of Ag to Pd inhibits the formation of Pd β-hydride due to the presence of Pd in a different form, most likely as an alloy. Thus, given the UV-Vis DR data, the presence of a negative peak below 90 °C confirms the formation of individual monometallic Ag and Pd NPs in the sample prepared by using the MeCN_IWI_H_2_ method. This is caused by the use of metal sources with a different nature in this method (silver nitrate and palladium acetylacetonate). The H_2_-TPR profile of the sample prepared by using the DS_N_2_H_4_ method demonstrates a broad positive peak of H_2_ consumption with a maximum at 176 °C (Figure 6) and can be attributed to the reduction of highly dispersed Ag–Pd alloy NPs [75]. The absence of hydrogen absorbance by the IWI_H_2_ sample can be connected with the low temperature of oxidative pretreatment [76] that is not sufficient for bulk oxidation of the relatively large NPs presented in this material.

### 3.3. Effect of Metal Loading

In order to investigate the effect of metal loading, the samples with the same Ag/Pd molar ratio of 1/1 and a total metal loading of 1–5%wt. were prepared and characterized. The UV-Vis DR analysis shows no formation of monometallic Ag NPs with increasing of the Ag–Pd content (Figure S5 in the SI). The increase in the metal loading leads to higher absorbance of irradiation in the visible light region. The surface area and micropore volume decrease as the metal content increases (Table S2 in SI), but not as dramatically as in comparison with the pristine UiO-66 (Table 2), which is similar to results for other types of NP@MOFs materials [24].

**Figure 7:**
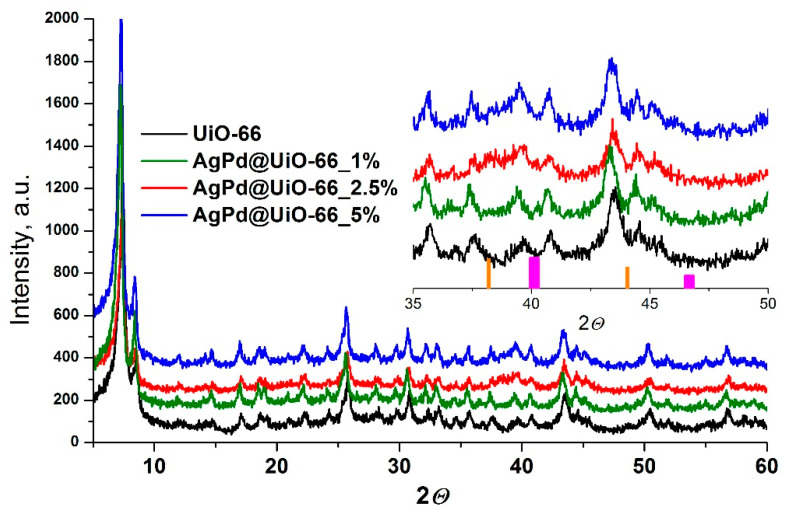
XRD pattern of UiO-66 (black) and AgPd@UiO-66 samples with 1 % (green), 2.5 % (red), and 5 % (blue) metal loading.

The PXRD pattern demonstrates that the UiO-66 structure remained unchanged even when 5% of metal was loaded (Figure 7).

The PXRD pattern for the DS_N_2_H_4_ AgPd@UiO-66 sample did not exhibit the characteristic reflections for Ag and Pd, thereby indicating the absence of crystalline monometallic Ag and Pd NPs. Only the reflections of the UiO-66 were observed in the PXRD pattern for the DS_N_2_H_4_ AgPd@UiO-66 sample with a metal loading of 1%wt. A further increase in the metal content resulted in the appearance of low-intensive broad reflections between the characteristic peaks of Ag(111) (2θ *=* 38.03°) and Pd(111) (2θ *=* 40.1°). This indicates the formation of highly dispersed Ag–Pd alloyed NPs with only a small fraction of crystalline bimetallic NPs with sizes above 3 nm. Figure S6 shows TEM images obtained from sample DS_N_2_H_4_ AgPd@UiO-66 with 5 wt.% of metals (1/1). Metallic NPs with sizes of ~8 nm are well seen on the external surface of UiO-66. Table S3 shows the close inspection of the XRD patterns to estimate the phase composition. The increase of the total metal loading from 1 to 5 %wt. did drastically not change the phase ratio on the samples prepared. Thus, the elaborated method of double solvent impregnation with the consequent reduction by hydrazine solution allows for preparing the immobilized bimetallic samples within the UiO-66 structure.

Figure S6 represents the SEM images of the AgPd@UiO-66 materials. The application of Ag and Pd nitrates as metal precursors led to the formation of uniform surface of the AgPd@UiO-66_ DS_N_2_H_4_ catalyst (Figure S6a,b). The impregnation with AgNO_3_ and Pd(AcAc)_2_ in acetonitrile followed by the reduction in a hydrogen flow (sample AgPd@UiO-66_MeCN_H2) resulted in the formation of large metal aggregates on the surface of the UiO-66 (Figure S6b). Metal immobilization technique had no effect on the morphology of the host material. This fact confirmed that the UiO-66 structure was stable during the immobilization process.

**Figure 8:**
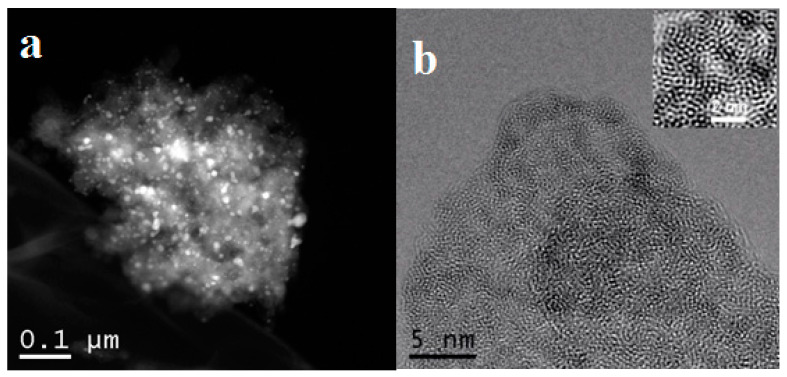
STEM-HAADF (**a**) and HRTEM (**b**), images of 5% AgPd@UiO-66 with an Ag/Pd molar ratio of 1/1.

The AgPd@UiO-66 sample with 5%wt. metal loading was characterized by using TEM and energy-dispersive spectroscopy (EDS). A dark field image indicates the presence of high electron density spots all over the sample that mostly have jagged edges and a diameter higher than the pore size of UiO-66 (Figure 8a).

However, it is well known that under electron-beam irradiation, a degradation of the MOF structure can occur, leading to agglomeration of the NPs that yields particles with sizes larger than in the as-prepared material [41]. The TEM and STEM-HAADF images show the degradation of the AgPd@UiO-66 sample comprising the changing of the structure of UiO-66 particles, as well as migration and coalescence of Ag–Pd particles. Ag–Pd particles with sizes of 2–10 nm were observed in the structure.

**Figure 9:**
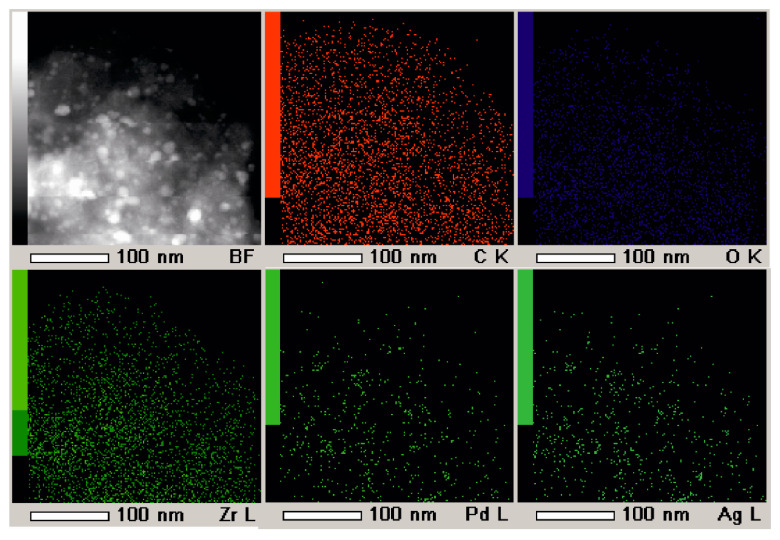
Energy-dispersive spectroscopy (EDS) images (map) of 5% AgPd@UiO-66 with a Ag/Pd molar ratio of 1/1.

At the same time, the bright-field TEM image at a larger scale confirmed the formation of metal NPs with a diameter about 1.5 nm (Figure 8b) inside the MOF cages. The FFT taken from the HR TEM images had a halo with a maximum corresponding to 0.29 nm. This parameter is characteristic of a strong reflection of tetragonal or cubic modifications of zirconia. The mesoporous support UiO-66 was restructured into nanostructured ZrO_2_, and the spacers between the clusters contained lighter functional groups linking the clusters. There are areas with higher concentrations of metal that can look like NPs with sizes exceeding pore sizes of UiO-66 at a small-scale image (Figure S7 in SI), but one cannot exclude the formation of a small fraction of bimetallic NPs on the external surface under high metal loading (Figure S7 in SI). The EDAX images confirmed a homogeneous three-dimensional distribution of the Ag–Pd NPs throughout the interior of the UiO-66 matrix (Figure 9).

The EDS taken from those sites with Ag–Pd NPs indicates the composition of intermetallide with the Ag/Pd ratio that depends on the size of the NPs. At the same time, the EDS images taken from the sites where Ag–Pd NPs are not observed in the STEM-HAADF mode do not contain intensive reflections of Ag and Pd. Atomically dispersed metal cannot be seen in the STEM-HAADF images due to the limitations of the resolution. However, the atoms of the metals can be the sites of ZrO_2_ cluster nucleation.

### 3.4. Catalytic Properties

The catalytic activity of AgPd/UiO-66 hybrid materials strongly depends on preparation procedures and nature of metal precursors. Firstly, the influence of the method of metallic NP immobilization in the UiO-66 on the basic directions of PG conversion was studied. The preparation method was shown to affect both PG conversion and selectivities towards the main products (entries 1–3, Table 4). The highest PG conversion (7%) was achieved for the AgPd/UiO-66 sample prepared by using the double solvent method (DS_N_2_H_4_) (entry 3). The main products were LA and HA. For the MeCN_WI_H_2_ sample, the PG conversion was very close to the one for DS_N_2_H_4_ sample, while the formation of AA as the main reaction product was observed (entry 2). The WI_H_2_ sample was inactive under the conditions used.

Application of MeCN solution of Pd(AcAc)_2_ and AgNO_3_ as metal sources mainly led to the formation of monometallic silver and palladium NPs in the composition of MeCN_WI_H_2_ sample, as is shown by the UV-Vis DR in Figure S2. The formation of AgPd alloyed NPs was observed for the DS_N_2_H_4_ sample prepared from water solution of silver and palladium nitrates as metal precursors. The same situation was observed for WI_H_2_ sample; however, the H_2_ reduction stage led to the formation of large bimetallic NPs located on the external surface of the support. It was concluded that the double solvent method with aqueous nitrate salts as precursors and N_2_H_4_ reduction was a favorable approach to prepare the hybrid AgPd bimetallic catalyst immobilized in the UiO-66 host structure.

Then the effect of Ag/Pd ratio in the composition of AgPd/UiO-66 hybrid materials with 1%wt. of metal loading on the catalytic properties in the PG oxidation was studied (entries 3–5, Table 4). All samples were prepared with the use of DS approach. The increasing of Ag content in bimetallic NPs (Ag/Pd = 85/15) led to the growth of selectivity towards LA (up to 80%) and HA (up to 15%) in comparison with the catalyst with Ag/Pd = 1/1 (entries 4 and 3, respectively) under similar PG conversions, while the Ag content reduction to Ag/Pd ratio = 15/85 was accompanied by both decreasing of the PG conversion and redistribution of products.

Finally, the influence of the metal loading from 0.5 to 5%wt. in the composition of AgPd/UiO-66 hybrid materials with Ag/Pd ratio amounting to 85/15 was investigated (entries 4, 7–9). The increasing of the bimetal content from 0.5 to up to 1.0%wt. in the composition of AgPd/UiO-66 sample with Ag/Pd = 85/15 led to PG conversion growth with the predominant formation of LA (selectivity was 64%) and HA (selectivity was 20%). Then upon further increase of bimetal loading, the PG conversion also increased to up to 10% and 12% for 3%wt. and 5%wt. bimetal contents, respectively, but the main product was HA with the selectivities of 30% and 41.7%, respectively and the selectivity towards LA decreased to up to 5%wt. and 2.5%wt.

An additional experiment was carried out to study the MGO conversion on UiO-66 sample without metal under comparable conditions (entry 10 in Table 4). It was shown that the HA conversion was 48% with the LA formation as the main product (77%). In addition, AA and FA were observed in reaction products as a result of the C–C bond cleavage in the intermediate molecules.

Low LA concentration in the reaction products can be associated with the decreasing of the accessibility of active acid-base sites of UiO-66 in AgPd/UiO-66 catalysts with 3% and 5% bimetal NPs due to a partial blocking of the porous space of the host material by large bimetal NPs located on the external surface. Table S2 shows that the surface area and micropore volume decrease as the metal loading increases. Moreover, the sizes of bimetal AgPd NPs increased for these catalysts (Figure 8a) and became equal to 2–10 nm. We believe that the minimal distance between the active redox and acid-base species (as close to each other as possible) is the key factor for the PG transformation into LA. In this context, the AgPd/UiO-66 catalyst with Ag/Pd ratio of 85/15 and 1%wt. loading of bimetal NPs prepared by the DS method from the nitrate salts as precursors is promising for the cascade PG transformation into LA under neutral media.

## 4. Conclusions

In this work, Ag–Pd@UiO-66 materials were synthesized by using different methods (wetness impregnation of silver or/and palladium precursors in water or acetonitrile solution, and the double solvent impregnation technique). Classical incipient wetness impregnation with an aqueous solution of silver and palladium nitrates followed by high-temperature reduction in hydrogen flow led to the formation of Ag–Pd alloyed NPs with diameters larger than the pore cages of the UiO-66. Replacement of the aqueous solution of metal precursors with acetonitrile and Pd(NO_3_)_2_ with palladium acetylacetonate in the same synthetic procedure resulted in the formation of individual Ag and Pd NPs on the external surface of the MOF. Only the modified double-solvent approach with one-pot reduction with hydrazine and the absence of drying step enabled us to obtain highly dispersed Ag–Pd alloy NPs located mostly inside the pores of UiO-66. The UiO-66 preserved its structure even after loading with as much as 5%wt. of metal. The obtained material possessed high concentrations of both Lewis acid and redox sites.

The catalytic properties of the new hybrid AgPd/UiO-66 catalysts prepared by employing different methods, with different Ag/Pd ratios and bimetal loading, were studied in oxidative propylene glycol transformation into lactic acid carried out in neutral reaction mixtures. Catalytic activity of the novel hybrid materials was found to depend strongly on the preparation procedure and nature of metal precursors. It was established that the double solvent method with aqueous nitrate salts as precursors and N_2_H_4_ reduction was a favorable approach to prepare a hybrid AgPd bimetallic catalyst immobilized in the UiO-66 host structure.

An increase in Ag content by up to 85% in the composition of bimetallic nanoparticles led to an increase in LA selectivity of up to 80% while retaining the PG conversion. The maximal PG conversion was reached at the Ag/Pd ratio of 1/1. Monometallic Pd or Ag/UiO-66 was almost inactive in PG transformation under tested conditions. Thus, the activity of the AgPd/UiO-66 materials in the catalytic PG oxidation is a result of synergistic interaction between the components in AgPd alloyed NPs. An increase of metal content in the AgPd/UiO-66 hybrid catalyst from 0.5% to up to 1%, with a Ag/Pd ratio of 85/15, led to enhanced PG conversion and LA selectivity. Further elevation of metal loading resulted in the formation of a fraction of AgPd NPs diameters above 3 nm, which were localized on the external surface of the MOF. The presence of active and more accessible AgPd NPs resulted in the highest PG conversion and HA selectivity but the selectivity towards LA became rather low. The AgPd/UiO-66 hybrid catalyst with a low metal content possessed a large fraction of small (<3 nm) NPs localized inside the UiO-66 structure. A close distance between the redox and Lewis acid sites in the limited space of UiO-66 facilitated PG transformation to LA in neutral media.

## Figures and Tables

**Table 1:** Methods of immobilization of Ag and Pd in UiO-66.

Abbreviation	Impregnation	Drying	Reduction Conditions
WI_H_2_	IWI	20 h at room temperature	H_2_/Ar at 200 °C 2 h
MeCN_WI_H_2_	IWI	20 h at room temperature	H_2_/Ar at 200 °C 2 h
DS_N_2_H_4_	DS	no drying	N_2_H_4_ at room temperature

**Table 2:** Textural characteristics of 1%Ag@UiO-66 prepared by using different methods.

Sample	UiO-66	WI_H_2_	MeCN_WI_H_2_	DS_N_2_H_4_
S_BET_, m^2^/g	1298	1268	1246	982
V_micropores_^a^, cm^3^/g	0.50	0.50	0.49	0.38

^a^—calculated by using the Horvath-Kawazoe sphere pore geometry method.

**Table 3:** Acid properties of UiO-66 and AgPd@UiO-66 (Ag/Pd = 1/1, 1%wt. metal loading) prepared by using DS_N_2_H_4._

Sample	Area under NH_3_-TPD Peak, a.u./g	Amount of Lewis Acid Sites, µmol/g
UiO-66	9.22	939
AgPd@UiO-66	8.32	847

**Table 4:** Catalytic activity of AgPd/UiO-66 in PG oxidation (T = 90 °C, P(O_2_) = 3 bar, reaction time = 7 h).

№	Preparation Method	Me NPs, wt %	Ag/Pd Ratio	PG/Me	PG Conv. %	LA Sel., %	HA Sel., %	AA Sel., %	FA Sel., %	Other Products* Sel., %
1	WI_H_2_	1	1/1	9174	1.0	20.0	5	0	6.7	68.7
2	MeCN_WI_H_2_	1	1/1	9174	5.0	0	0	75	8.3	16.7
3	DS_N_2_H_4_	1	1/1	9174	7	35.7	10	0	17.6	36.7
4	DS_N_2_H_4_	1	85/15	9174	6	80	15	0	2.8	2.2
5	DS_N_2_H_4_	1	15/85	9174	2	9	15	25	13.3	37.7
6	DS_N_2_H_4_	1	0/1	9174	1	0	0	0	0	100
7	DS_N_2_H_4_	0.5	85/15	18,348	2.5	64	20	0	4.5	11.5
8	DS_N_2_H_4_	3	85/15	3058	10	5	30	4	4.7	56.3
9	DS_N_2_H_4_	5	85/15	1834	12	2.5	41.7	8.7	1.4	54.3
10	UiO-66	0	0	300 **	48	77	0	10	5	8

LA—lactic acid, HA—hydroxyacetone, AA—acetic acid, FA—formic acid. *other products—MGO, CH_2_O, CO_2_, **—PG/Zr ratio.

## Supplementary Materials

The following are available online at https://www.mdpi.com/1996-1944/13/23/5471/s1, Figure S1: XRD pattern of UiO-66, Figure S2: FT-IR spectrum of UiO-66, Figure S3: TGA of as-prepared DS_N_2_H_4_ 1%Ag,Pd@UiO-66, Figure S4: UV-Vis DR spectra of Ag,Pd@UiO-66 prepared by using different methods, Figure S5: UV-Vis DR spectra of Ag,Pd@UiO-66 with 1%wt. (blue), 2.5%wt. (black), and 5%wt. (red) metal loading, Figure S6: SEM image of 1% Ag-Pd@UiO-66 prepared by using different techniques: (a) IWI_H_2_, (b) MeCN_H_2_, and (c) DS_N_2_H_4_, Figure S6: TEM images of 5% Ag-Pd@UiO-66 with an Ag/Pd molar ratio of 1/1, Table S1: Phase compositions of Ag@UiO-66 samples prepared by using different methods, Table S2: Textural characteristics of Ag/UiO-66 prepared by DS_N_2_H_4_ with different metal loading, Table S3: Phase compositions of Ag,Pd@UiO-66 samples with different metal loading.
