# Supplementary material for: Bimetallic AgPd/UiO-66 Hybrid Catalysts for Propylene Glycol Oxidation into Lactic Acid
Source: Materials (Basel). 2020 Nov 30;13(23):5471. doi: 10.3390/ma13235471 (PMC7731450; doi:10.3390/ma13235471)
Supplement: Supplementary file 1 [file materials-13-05471-s001.pdf]

materials

Supplementary Materials: Bimetallic AgPd/UiO-66 hybrid catalysts for propylene glycol oxidation into lactic acid

Sergey Ten [1], Viktoriia V. Torbina [1], Vladimir I. Zaikovskii [2], Sergei A. Kulinich [3,4], and Olga V. Vodyankina [1,*]

[1] Tomsk State University, 36 Lenin ave., Tomsk 634050, Russia
[2] Boreskov Institute of Catalysis, 5, Lavrentiev ave., Novosibirsk 630090, Russia
[3] Tokai University, Research Institute of Science & Technology, Kanagawa 259-1292, Japan
[3] Far Eastern Federal University, Vladivostok 690091, Russia
* *Correspondence:* O.V. Vodyankina (vodyankina_o@mail.ru)

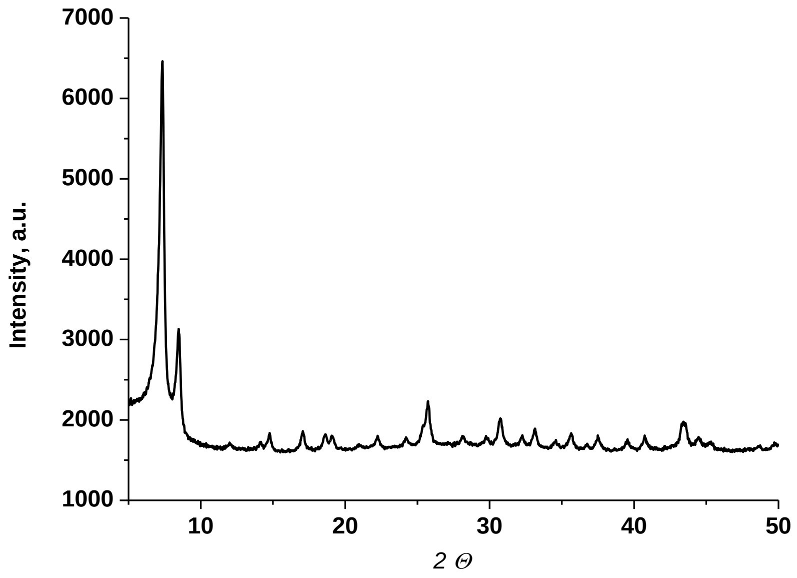

Figure 1. XRD pattern of UiO-66.

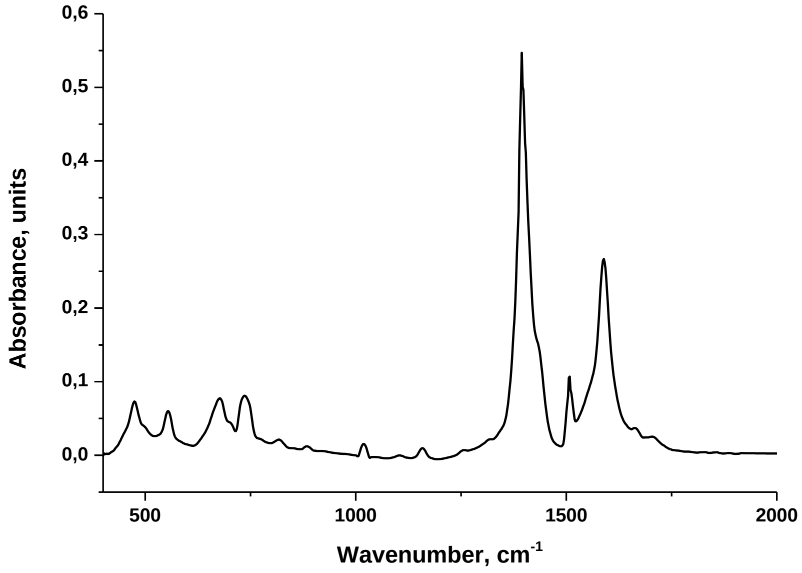

Figure 2. FT-IR spectrum of UiO-66.

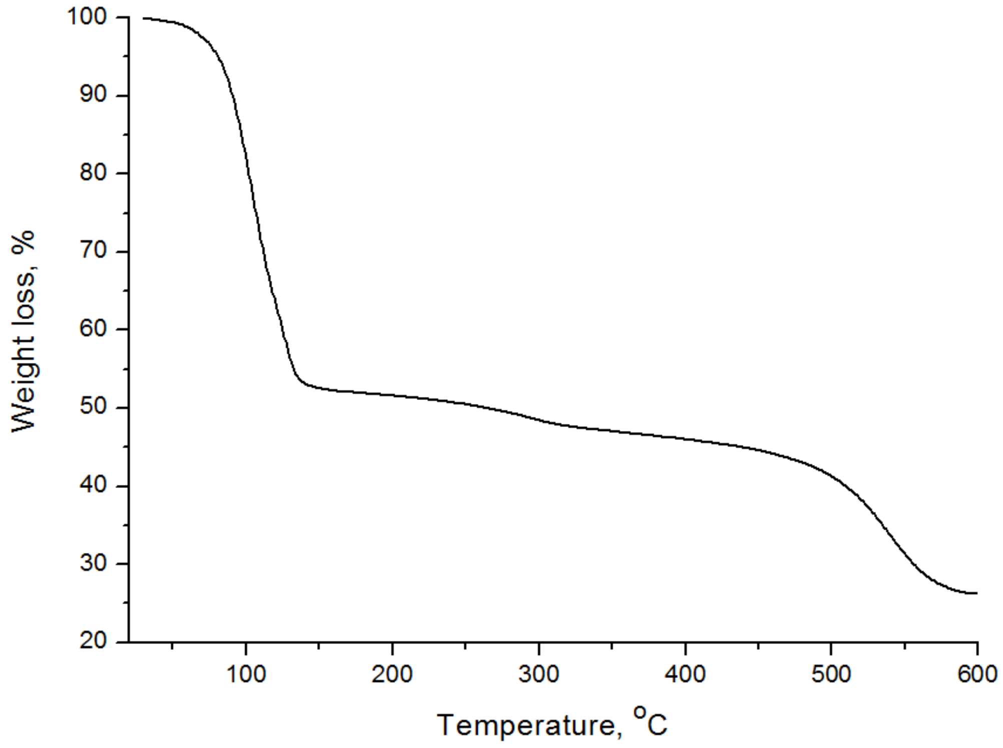

Figure 3. TGA of as-prepared DS_N_2H_4 1%Ag,Pd@UiO-66.

Preparation method	Metal loading, wt %	Estimation of phase composition according to Jana2006, wt%		
		UiO-66	ZrO_2	Ag
**MeCN_H_2**	1	92.7	6	1.4
DS_N_2H_4	1	95.1	5	0.2
DS_H_2	1	85.1	11	4.1

Table 1. Phase compositions of Ag@UiO-66 samples prepared by different methods.

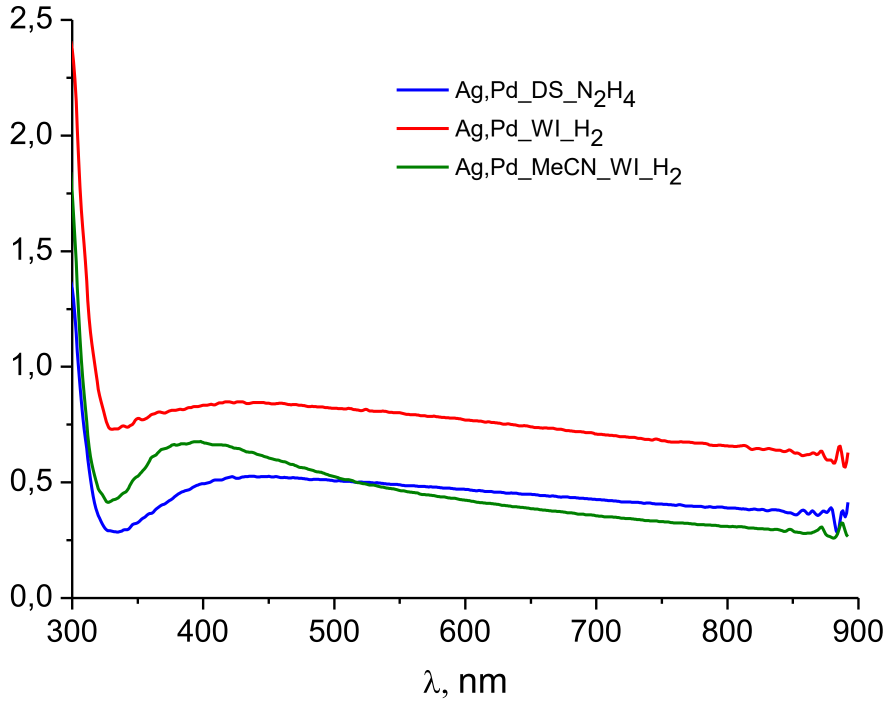

Figure 4. UV-Vis DR spectra of Ag,Pd@UiO-66 prepared by different methods.

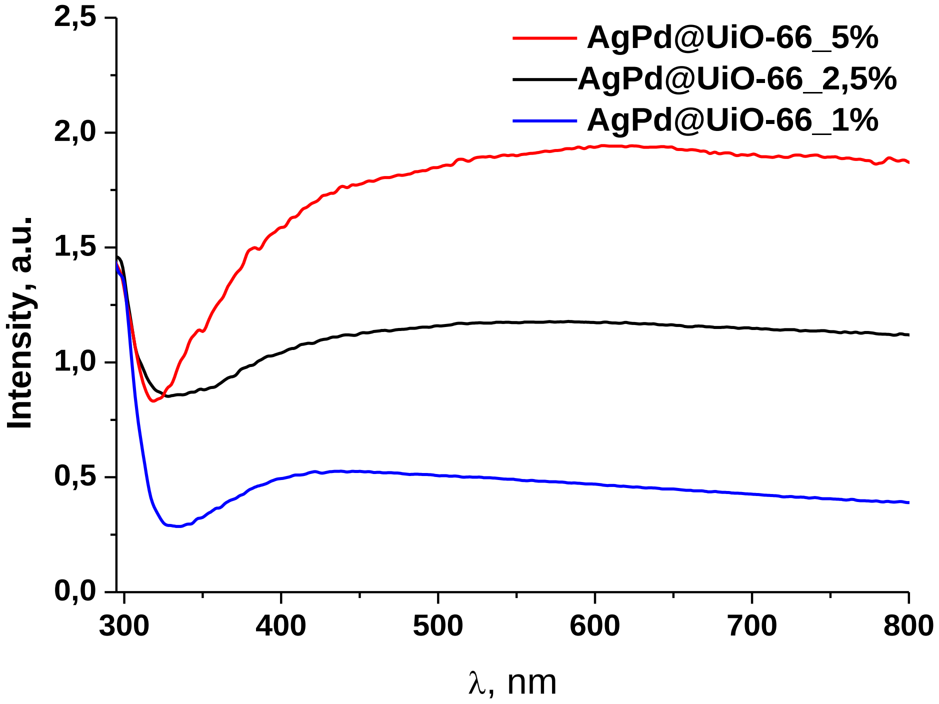

Figure 5. UV-Vis DR spectra of Ag,Pd@UiO-66 with 1%wt. (blue), 2.5%wt. (black) and 5%wt. (red) metal loading.

Table 2. Textural characteristics of Ag/UiO-66 prepared by DS_N_2H_4 with different metal loading.

Metal loading, %	S_{BET}, m²/g	$V_{micropores}^a$, cm³/g
1	982	0.38
2.5	917	0.37

5	903	0.36

[a] – calculated by Horvath-Kawazoe sphere pore geometry method.

Table 3. Phase compositions of Ag,Pd@UiO-66 samples with different metal loading.

**Ag/Pd ratio in bimetallic samples**	Metal loading, wt%	**Estimation of phase composition of samples according to Jana2006 software, wt%**			
		UiO-66	ZrO_2	Ag	Pd
1:1	1	94.4	5	0.3	0.6
1:1	2.5	89.9	7	2.0	0.6
1:1	5	93.1	6	0.4	0.5

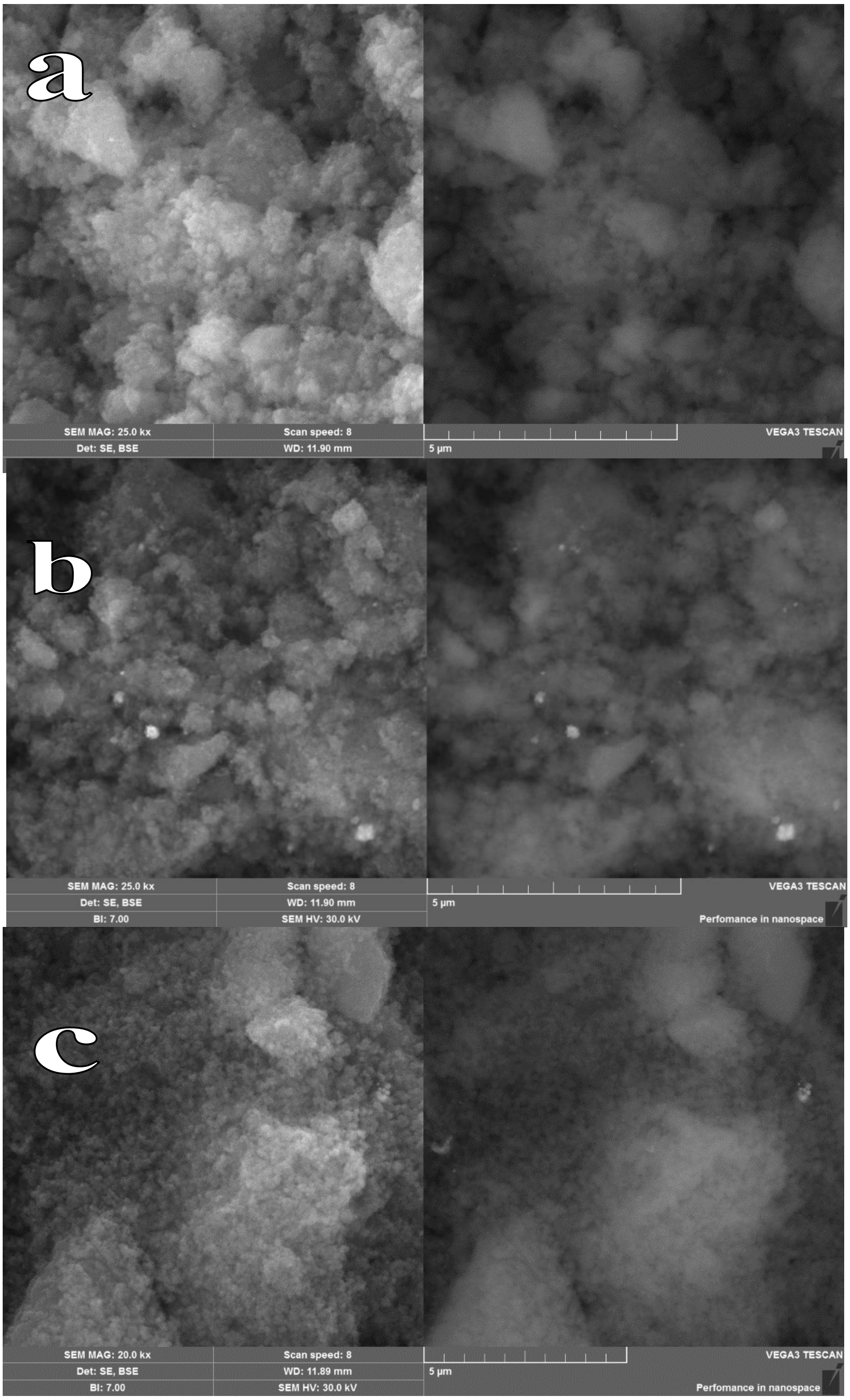

Figure S6. SEM image of 1% Ag-Pd@UiO-66 prepared by different techniques: (**a**) IWI_H_2, (**b**) MeCN_H_2, (**c**) DS_N_2H_4.

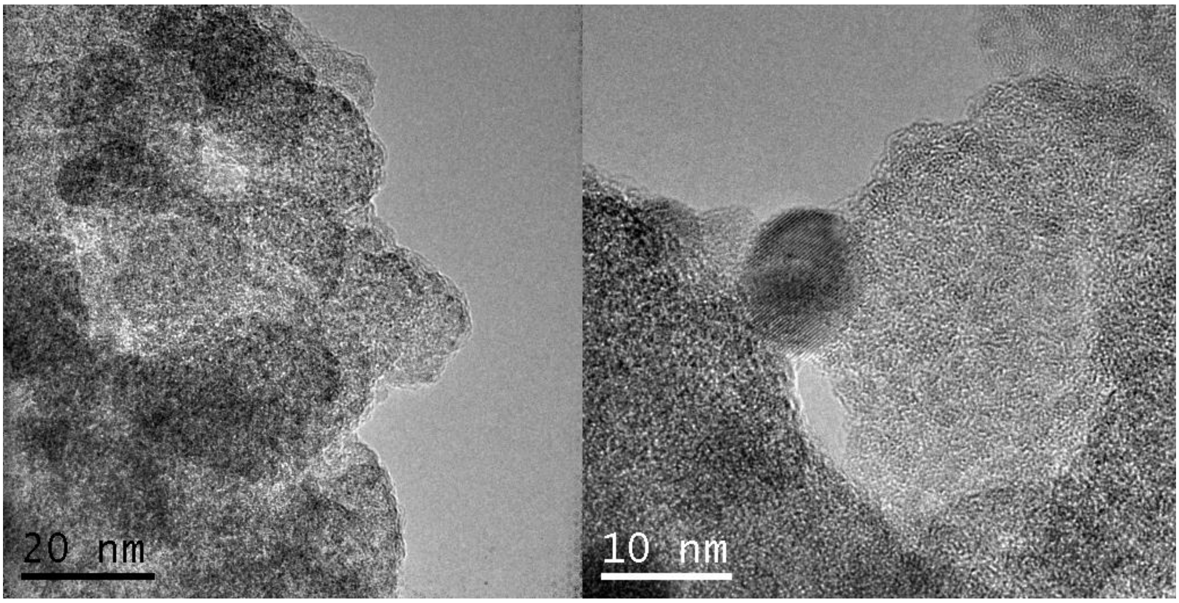

Figure 7. TEM images of 5% Ag-Pd@UiO-66 with Ag/Pd molar ratio of 1/1.

© 2020 by the authors. Licensee MDPI, Basel, Switzerland. This article is an open access article distributed under the terms and conditions of the Creative Commons Attribution (CC BY) license (http://creativecommons.org/licenses/by/4.0/).
